# The impact of ultraviolet- and infrared-based laser microdissection technology on phosphoprotein detection in the laser microdissection-reverse phase protein array workflow

**DOI:** 10.1186/s12014-020-09272-z

**Published:** 2020-03-09

**Authors:** Allison L. Hunt, Mariaelena Pierobon, Elisa Baldelli, Julie Oliver, Dave Mitchell, Glenn Gist, Nicholas W. Bateman, G. Larry Maxwell, Emanuel F. Petricoin, Thomas P. Conrads

**Affiliations:** 1 Women’s Service Line, Inova Health System, 3300 Gallows Rd., Falls Church, VA 22042 USA; 2 Gynecologic Cancer Center of Excellence, Department of Obstetrics and Gynecology, Uniformed Services University and Walter Reed National Military Medical Center, 8901 Wisconsin Avenue, Bethesda, MD 20889 USA; 3 Center for Applied Proteomics and Molecular Medicine, George Mason University, Manassas, VA USA; 4 The Henry M. Jackson Foundation for the Advancement of Military Medicine, Inc., 720A Rockledge Drive, Suite 100, Bethesda, MD 20817 USA; 5 3289 Woodburn Rd, Suite 375, Annandale, VA 22003 USA

**Keywords:** Phosphoprotein, Laser microdissection, Laser capture microdissection, Reverse phase protein array, Proteomics

## Abstract

Reversible protein phosphorylation represents a key mechanism by which signals are transduced in eukaryotic cells. Dysregulated phosphorylation is also a hallmark of carcinogenesis and represents key drug targets in the precision medicine space. Thus, methods that preserve phosphoprotein integrity in the context of clinical tissue analyses are crucially important in cancer research. Here we investigated the impact of UV laser microdissection (UV LMD) and IR laser capture microdissection (IR LCM) on phosphoprotein abundance of key cancer signaling protein targets assessed by reverse-phase protein microarray (RPPA). Tumor epithelial cells from consecutive thin sections obtained from four high-grade serous ovarian cancers were harvested using either UV LMD or IR LCM methods. Phosphoprotein abundances for ten phosphoproteins that represent important drug targets were assessed by RPPA and revealed no significant differences in phosphoprotein integrity from those obtained using higher-energy UV versus the lower-energy IR laser methods.

## Background

Laser microdissection (LMD) and laser capture microdissection (LCM) enable target populations of cells to be selectively harvested from heterogeneous admixtures of cells in the tissue microenvironment (TME), either from histological fresh-frozen or formalin-fixed paraffin-embedded (FFPE) tissue thin sections [1–3]. The ability to harvest and enrich homogenous cell populations and/or remove undesired tissue regions using LMD is broadly enabling to numerous analytical workflows for biomedical research. One such application is for cancer research as the cancer TME comprises a highly varied admixture of non-tumor cells, including fibroblasts, infiltrating lymphocytes, macrophages, along with fibrotic and/or necrotic regions. Thus, it is becoming increasingly evident that biomolecular analyses of each of these cell populations is important to make new strides in our understanding of this complex cellular ecosystem. This workflow is likely to lead to new advances in multi-omic analyses of cancer tissues, including proteomics, particularly since analysis of proteins extracted from whole tissue specimens limits detection of disease-related proteins due to the heterogeneous mixture of tumor with non-tumor cells [4–10]. Studies employing LMD as part of pre-analytical preparation of samples have identified cancer-associated molecular changes and prognostic biomarkers for many cancers including ovarian cancer [11–28], cervical cancer [29–31], vulvar cancer [32, 33], and uterine cancer [34–37]. Many cancer signaling pathways regulating cell growth, proliferation, differentiation, and metastasis are mediated by the concerted actions of kinases and phosphatases and are frequently disrupted or dysregulated in cancer (reviewed in [38–40]). It is therefore critically important that pre-analytical preparation of specimens for phosphoproteomic analysis does not disrupt this labile post-translational modification, including collection of tissue samples by LMD. In practice, the two general laser microscopy platforms differ mechanistically in the way cells are procured, namely via low energy infrared (IR) contact laser-capture microdissection (LCM) [1, 2] or by high energy ultraviolet (UV) cutting LMD [3, 41–44].

The IR LCM and UV LMD platforms each rely on light microscopy to visualize and identify target cell populations for harvest from tissue sections. The IR LCM platform uses a thermolabile polymer film containing ethylene vinyl acetate impregnated with a dye that absorbs light at near-IR wavelengths which is brought into physical contact with the tissue section on a glass slide [1, 2]. The film is situated in a plastic support cap that optically focuses the laser in the same plane as the tissue section. An IR laser heats the film where it contacts the selected target regions; cells in contact with the heated polymer attach and are sheared from the remaining tissue. An extraction buffer is used to free the embedded cells from the polymer for subsequent molecular analysis. In contrast, the UV platform employs polyethylene napthalate (PEN), polyethylene tetraphthalate (PET) or polyphenylene sulfide (PPS) [3, 41, 42] membrane slides onto which thin tissue sections are cut. Target cells are harvested through the action of a UV laser that cuts the selected membrane and tissue elements that are collected by gravity into a collection vessel located below the slide or are catapulted by a laser pulse into a cap above the slide.

In the case of IR LCM, the dye within the film absorbs IR light to reduce damage to cellular components. Additionally, the IR laser typically used is low in energy and instantaneously applied in a pulsed fashion to further minimize IR-induced cell damage [1]. However, because the harvested tissue is effectively “melted” onto the LCM membrane, efficient cellular lysis and recovery of biomolecules requires harsh detergents that can be problematic for some downstream chemical manipulations and/or analytical applications, such as mass spectrometry. UV LMD harvests represent substantial versatility because tissue recovery does not involve melting of a membrane to the tissue. Some concern remains, however, regarding whether UV LMD provides a comparably suitable acquisition technique for analyzing labile macromolecules such as phosphoproteins in histologically prepared samples because of the potential for higher energy UV exposure to cells and molecules in direct field proximity to the laser. Analysis of clinical samples by IR LCM coupled to reverse-phase protein array (RPPA) has generated highly accurate and reliable phosphoprotein data from FFPE and frozen tissues [6, 8, 27, 45–48]. Conversely, staining and routine sample processing following LMD (aside from microdissection itself) have been shown to negatively impact a variety of molecular analyses, including phosphoprotein levels in frozen tissues [49], as well as RNA integrity [50, 51] and the availability and/or resolution of proteins and phosphoproteins [45, 52–56]. UV LMD has recently been proposed as faster and more precise than IR LCM as a preparatory tool for certain types of molecular analyses [57].

This study aimed to assess whether there is a measurable difference between UV- and IR-mediated laser microdissection on phosphoprotein integrity by analyzing key cancer phosphoprotein abundances by RPPA from four high grade serous epithelial ovarian carcinoma (HGSOC) specimens. Our results demonstrate that there is no significant difference between UV- and IR-mediated laser microscopy on phosphoprotein integrity.

## Materials and methods

### Tissue specimens

Snap-frozen tissue specimens were obtained from four ovarian high grade serous ovarian cancer (HGSOC) patients within 30 min of surgical resection under an IRB exempt protocol (Western IRB approved). Two consecutive thin tissue sections (10 µm) embedded in optimal cutting temperature (OCT) medium (Fisher Scientific) were cut by microtome from each patient specimen. One section from each specimen was placed on PEN membrane slides (Leica Microsystems) for UV LMD and the other was placed on uncharged glass slides (Premium Glass Microscope Slides, Daigger) for IR LCM. Tissue sections were imaged before and after UV LMD or IR LCM using an Aperio AT2 digital whole slide scanner (Leica).

### Histologic staining

Slides were thawed and stained immediately prior to laser microscopy harvest. One glass and one PEN membrane slide prepared from consecutive tissue slices were stained for IR LCM and UV LMD, respectively for downstream RPPA analysis. Slides were fixed in 70% ethanol, rehydrated in deionized water, stained using Mayer’s Hematoxylin, and rinsed using deionized water and Scott’s Tap Water (Thermo Fisher Scientific). After staining and color development, slides were dehydrated in graded ethanol washes with two final rinses in xylene. Protease inhibitors (Roche) were added to all solutions except for the 100% ethanol and xylene washes.

### Laser microdissection

**Fig. 1:**
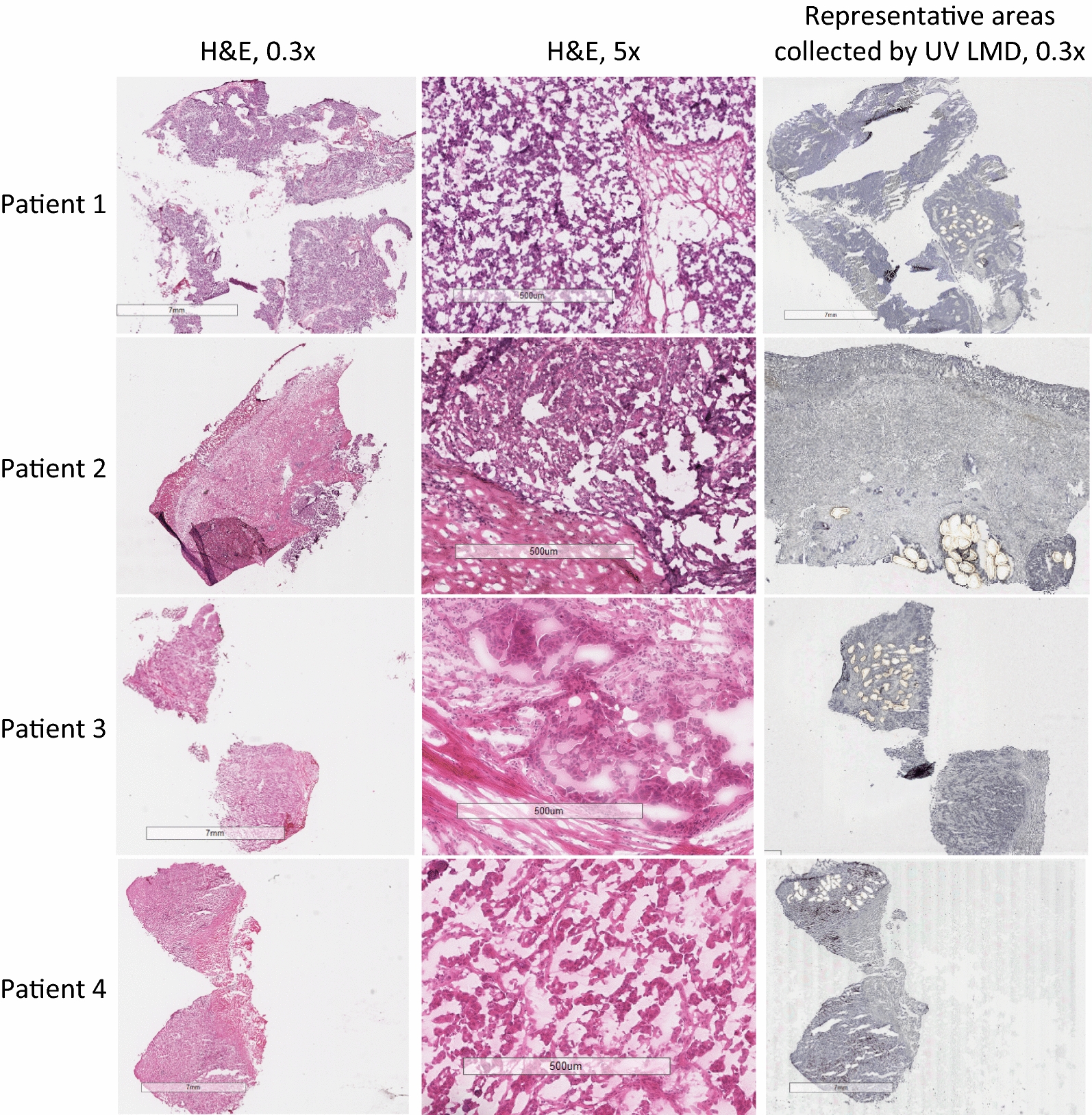
Micrographs of HGSOC tissue sections before and after tumor epithelial cell harvest by laser microdissection. A representative H&E-stained section for each patient (n = 4) is shown at 0.3× (1st column) and 5× (2nd column) magnification. Tumor epithelium from tissue thin sections (10 µm) was enriched via ultraviolet laser microdissection (UV LMD) or infrared laser capture microdissection (IR LCM). Representative areas following microdissection of tumor epithelium are shown at 0.3× magnification (3rd column); the images shown are post-UV LMD

IR LCM and UV LMD were performed independently on one consecutive tissue section each from each patient. UV LMD was performed on a LMD7 (Leica Microsystems) and IR LCM was performed on a PixCell II system (Arcturus). Two mm^2^ of tumor epithelium (yielding a final diluted protein concentration of approximately 0.25 µg/µl) from each slide was microdissected within 30 min for RPPA. A separate Hematoxylin and Eosin (H&E)-stained slide was used as a reference to map tissue zones for tumor epithelium collection by both LMD techniques (Fig. 1).

For UV LMD, the 2 mm^2^ of tumor epithelium was collected into a dry tube to which the extraction/lysis buffer consisting of a 1:1 mixture of Tissue Protein Extraction Reagent (T-PER; Pierce), Novex 2× Tris–Glycine SDS Sample Buffer (Invitrogen), and 2.5% *v/v* 2-mercaptoethanol was added. Collected tissue was briefly centrifuged and frozen at − 80 °C. For IR LCM, protein extraction was performed as previously described [46]. The LCM caps were visually examined for tissue debris or non-specific tissue adhesion, which was removed by blotting the cap with a CapSure cleanup pad (Arcturus). In brief, LCM caps and LMD tubes were similarly incubated with extraction buffer, cell lysates were then collected and boiled for 10 min before storage at − 80 °C.

### Reverse phase protein microarray

After thawing, all lysates for RPPA analysis were heated at 100 °C for 2 min in a dry heat block, cooled to ambient temperature, centrifuged, and used for printing microarrays. Using a 2470 Aushon Arrayer (Aushon BioSystems), samples were immobilized onto nitrocellulose-coated glass slides (Grace Biolabs) in technical triplicates as previously described [46]. Selected arrays were stained with Sypro Ruby Protein Blot Stain (Molecular Probes), according to manufacturer’s instructions to estimate the total amount of protein in each sample [46].

Before immunostaining, remaining slides were treated with Reblot Plus Mild Antibody stripping solution (Chemicon) for 15 min at room temperature, washed twice with PBS, and incubated in I-block solution (Tropix) for at least 4 h. Immunostaining was performed on an automated system (Dako) and each array was probed with one antibody targeting a protein of interest. Samples were probed with a total of ten antibodies targeting the phosphorylated forms of Akt S473 (Cell Signaling catalog #9271; 1:100), c-Abl T735 (Cell Signaling catalog #2864; 1:50), EGFR Y1068 (Cell Signaling catalog #2234; 1:50), HER2 Y1248 (Imgenex catalog #90189-1; 1:500), HER3 Y1289 (Cell Signaling catalog #4791; 1:200), ERK1/2 T202/Y204 (Cell Signaling catalog #9101; 1:1000), p70S6K T389 (Cell Signaling catalog #9205; 1:100), PDGFR Y751 (Cell Signaling catalog #3161; 1:50), Rb S780 (Cell Signaling catalog #3590; 1:2000), and RET Y905 (Cell Signaling catalog #3221; 1:100). Antibody specificity and linear dynamic range were previously tested [58]. Samples were then incubated with a secondary biotinylated goat anti-rabbit (Vector Laboratories; 1:7500) and with the commercially available tyramide-based Catalyzed Signal Amplification System (CSA, Dako) coupled with a fluorescent streptavidin-conjugated IRDye680 dye (LI-COR Biosciences). One slide was probed with secondary antibody only and used as a negative control for normalization purposes.

Stained arrays were scanned with a laser PowerScanner (TECAN) using the appropriate wavelength channel. Image analysis was performed using a commercially available software (MicroVigene v5.1.0.0, VigeneTech, Inc.). The software automatically performs spot finding, subtraction of local background and of nonspecific signal collected through the negative control slide(s). Samples were then normalized to the amount of protein and averaged across replicates.

## Results

**Fig. 2:**
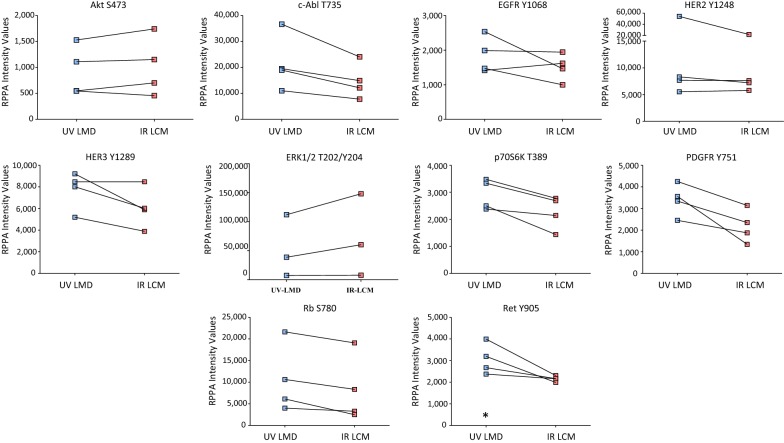
Phosphoprotein abundances in laser microdissected ovarian cancer tumor epithelial cells assessed by reverse-phase protein array. ERK1/2 pT202/pY204 in the UV LMD enriched Patient 1 sample is reported at complete signal saturation (150,000 RFU). The asterisk (*) indicates a significant difference (p < 0.05) in rank orders determined by a non-parametric Mann–Whitney U Test between matched ultraviolet laser microdissection (UV LMD) and infrared laser capture microdissection (IR LCM) collections

**Fig. 3:**
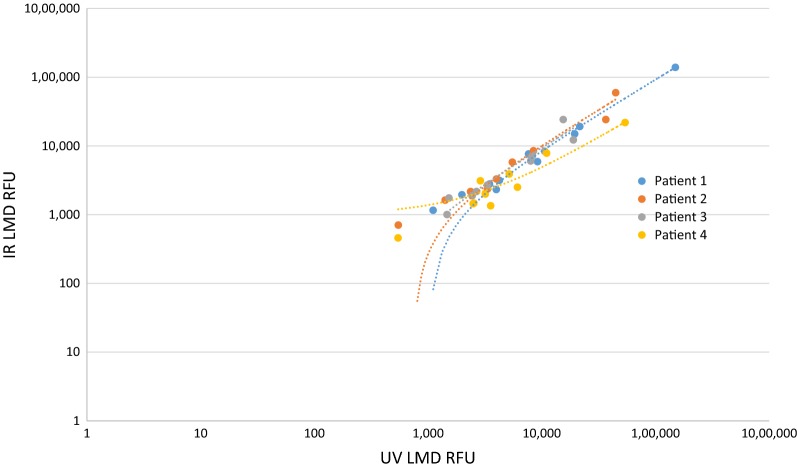
Correlation of ten phosphoproteins from high grade serous ovarian cancer tumor epithelium after laser microdissection. Fluorescence measurements (RFU) of phosphoprotein abundances from four patients enriched via ultraviolet laser microdissection (UV LMD) (x-axis) or infrared laser capture microdissection (IR LCM) (y-axis). ERK1/2 pT202/pY204 in the UV LMD enriched Patient 1 sample was measured at complete signal saturation (150,000 RFU) and was therefore excluded from this figure as to not impact the correlation values

Tumor epithelial cells were harvested using UV LMD or IR LCM from consecutive snap-frozen HGSOC patient tumor tissue thin sections (n = 4) (Fig. 1). The levels of ten key phosphoproteins involved in PI3K/AKT/mTOR signal transduction and related pathways which are frequently activated in ovarian cancer [59, 60] were analyzed using a standardized analytical panel of antibodies [58] (Additional file 1: Table S1). Comparative analyses revealed that the abundance level of all phosphoproteins remained consistent between the UV- and IR-mediated harvests (Fig. 2). A non-parametric Mann–Whitney-based comparison between matched UV LMD and IR LCM indicated that rank orders were not statistically different for the measured phosphoproteins, with the exception of pRET Y905 (*p *= 0.0286, Table 1). Pearson and Spearman correlations among all phosphoprotein abundances measured per patient confirmed high concordance between samples microdissected by both techniques (Fig. 3, Table 2). The Pearson *r* values were 0.9996 (*p *= 8.647 × 10^−13^), 0.9393 (*p *= 2.838 × 10^−07^), 0.8459 (*p *= 6.213 × 10^−05^), and 0.9847 (*p *= 3.423 × 10^−09^) for Patients 1–4, respectively. Similarly, Spearman’s rho (ρ) values showed high correlation and were 0.9890, 0.9941, 0.9934, and 0.9216 for Patients 1–4, respectively.

**Table 1:** Confidence levels for the ten phosphoprotein abundances assessed by reverse phase protein array

Phosphosite(s)	p-value
Akt S473	0.8857
cAbl T735	0.4857
EGFR Y1068	0.4857
Erb2 Y1248	0.6857
Erbb3 Y1289	0.4000
ERK1/2 T202/Y204	0.7000
p70s6 T389	0.3429
PDGFR Y751	0.0571
Rb S780	0.4857
Ret Y905	0.0286*

The asterisk (*) indicates significant difference (p < 0.05) in rank orders determined by a non-parametric Mann–Whitney U Test between matched ultraviolet laser microdissection (UV LMD) and infrared laser capture microdissection (IR LCM) collections of tumor epithelial cells from four high grade serous ovarian cancer patients. The ERK1/2 pT202/pY204 in the UV LMD enriched Patient 1 sample was measured at complete signal saturation (150,000 RFU), thus the Mann–Whitney p-value reported for ERK pT202/pY204 was calculated only using Patients 2–4

**Table 2:** Pearson and Spearman correlations for all phosphoprotein abundances assessed by reverse phase protein array

	Pearson r-value	p-value	Spearman’s Rho
Patient 1	0.9867	9.794E−11	0.9852
Patient 2	0.9393	2.838E−07	0.9941
Patient 3	0.8459	6.213E−05	0.9934
Patient 4	0.9847	3.423E−09	0.9216

Pearson correlation coefficients (r-values) and associated p-values from a two-tailed Student t-distribution are reported for all phosphoprotein abundances (n = 10 for Patients 2–4; n = 9 for Patient 1) from each patient (n = 4) measured by RPPA after ultraviolet laser microdissection (UV LMD) and infrared laser capture microdissection (IR LCM). The ERK1/2 pT202/pY204 in the UV LMD enriched Patient 1 sample was measured at complete signal saturation (150,000 RFU) and was therefore excluded from these calculations

## Discussion

In this study we conducted a first-of-its kind comparison between two popular cellular isolation techniques that are being extensively used in a number of important precision oncology programs such as the U.S. Department of Defense APOLLO program [61–63] and the I-SPY2 TRIAL series. Several prior precision studies have compared reproducibility, sampling heterogeneity, and laser effects for the LCM process, as well as accuracy studies comparing LCM generated HER2 and activated HER2 to FISH and IHC [8, 47, 48, 64, 65]. These previous studies have already demonstrated the precision and accuracy of the underpinning microdissection-RPPA workflow. Here we demonstrate that UV or IR-mediated laser microdissection does not significantly impact the quality of phosphoprotein analysis by RPPA. We found that the abundance of each phosphoprotein measured for the four HGSOC patient specimens tested was highly concordant in samples collected using either UV LMD or IR LCM techniques. Phosphoprotein levels in tissue sections microdissected with UV LMD were slightly higher in 31/40 (78%) of the comparisons. It is emphasized that this trend was not significant and that the results from each technique were highly correlated (with all *p *< 6.213 × 10^−05^). Thus, phosphoprotein abundances may be marginally improved for the analytes measured when microdissected using UV LMD, albeit these results did not achieve significance.

The choice of UV LMD and IR LCM will ultimately depend on the experimental aims and the heterogeneity of the tissue microenvironment itself. UV LMD confers ease of use and flexibility for sample collection, including higher sample processivity and is well suited for capturing large areas of relatively homogeneous cellular regions [57]. The non-contact UV LMD method allows for tissue from multiple sections or slides to be collected into the same tube, minimizing potential for sample loss. The current polymer caps used in IR LCM have a finite surface area, thus larger tissue sections or multiple tissue sections require additional caps for collection. This platform allows for direct capture of defined cellular regions, single cells and cell layers, and intermingled complex cellular microenvironments that could be present in any given tissue sample. Using this approach, another important hallmark is the ability to directly visualize and conduct pathology review of the cells captured without the need of complex difference imaging of regions collected before and after harvest, which is currently required for secondary pathology review of tissue regions harvested by UV LMD. The buffer necessary for extracting cells from the polymer caps however contain reagents that are incompatible with some downstream analytical approaches, including mass spectrometry (MS). Additional sample preparation steps such as filter-aided sample preparation (FASP) are required to remove the MS-incompatible reagents [66] from samples enriched using the current IR LCM caps. By comparison, the non-contact UV LMD method allows for a variety of buffer types to be added into the collection tube after LMD enrichment allowing for its incorporation into the standardized workflows of multiple analytical techniques.

## Conclusions

Our analysis demonstrates no significant negative impact on phosphoprotein recovery using high energy UV LMD versus IR LCM enrichment as measured by RPPA analysis. Further analysis on an expanded number of phosphoproteins as well as evaluating UV LMD vs IR LCM in the context of analysis of smaller isolated cellular regions will be the focus of future studies.

## Supplementary information

**Additional file 1: Table S1.** Normalized reverse phase protein array abundance values. The asterisk (*) indicates complete signal saturation for the ERK1/2 pT202/pY204 measured in the UV LMD enriched Patient 1 sample.

## Data Availability

Not applicable.

## Abbreviations

LMD: Laser microdissection
LCM: Laser capture microdissection
TME: Tissue microenvironment
FFPE: Formalin-fixed paraffin-embedded
IR LCM: Infrared contact laser capture microdissection
UV LMD: Ultraviolet cutting laser microdissection
PEN: Polyethylene napthalate
PET: Polyethylene tetraphthalate
PPS: Polyphenylene sulfide
RPPA: Reverse-phase protein array
HGSOC: High grade serous ovarian carcinoma
OCT: Optimal cutting temperature
MS: Mass spectrometry
FASP: Filter-aided sample preparation
Akt: Protein kinase B
cAbl: Tyrosine kinase ABL
EGFR: Epidermal growth factor receptor
HER2: Human epidermal growth factor receptor 2
HER3: Human epidermal growth factor receptor 3
ERK1/2: Extracellular signal-regulated kinase 1/2
P70S6K: Ribosomal protein S6 kinase
PDGFR: Platelet derived growth factor receptor
Rb: Retinoblastoma
RET: Rearranged during transcription
PI3K: Phosphoinositide 3-kinase
mTOR: Mammalian target of rapamycin
