# Supplementary material for: The impact of ultraviolet- and infrared-based laser microdissection technology on phosphoprotein detection in the laser microdissection-reverse phase protein array workflow
Source: Clin Proteomics. 2020 Mar 9;17:9. doi: 10.1186/s12014-020-09272-z (PMC7061469; doi:10.1186/s12014-020-09272-z)
Supplement: Supplementary file 1 — Additional file 1: Table S1. Normalized reverse phase protein array abundance values. The asterisk (*) indicates complete signal saturation for the ERK1/2 pT202/pY204 measured in the UV LMD enriched Patient 1 sample. [file 12014_2020_9272_MOESM1_ESM.pptx]

## Slide 1
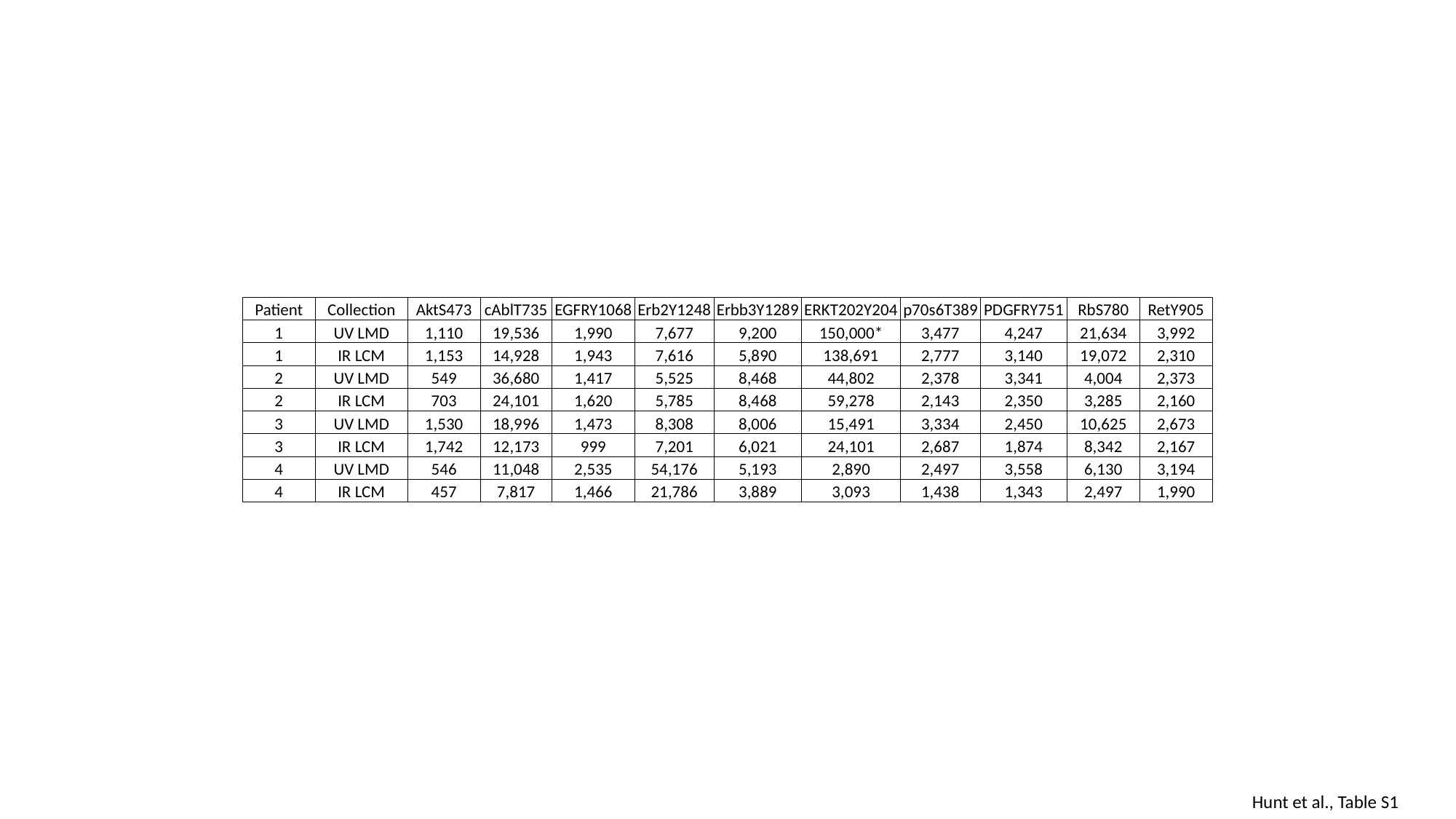

| Patient | Collection | AktS473 | cAblT735 | EGFRY1068 | Erb2Y1248 | Erbb3Y1289 | ERKT202Y204 | p70s6T389 | PDGFRY751 | RbS780 | RetY905 |
| --- | --- | --- | --- | --- | --- | --- | --- | --- | --- | --- | --- |
| 1 | UV LMD | 1,110 | 19,536 | 1,990 | 7,677 | 9,200 | 150,000\* | 3,477 | 4,247 | 21,634 | 3,992 |
| 1 | IR LCM | 1,153 | 14,928 | 1,943 | 7,616 | 5,890 | 138,691 | 2,777 | 3,140 | 19,072 | 2,310 |
| 2 | UV LMD | 549 | 36,680 | 1,417 | 5,525 | 8,468 | 44,802 | 2,378 | 3,341 | 4,004 | 2,373 |
| 2 | IR LCM | 703 | 24,101 | 1,620 | 5,785 | 8,468 | 59,278 | 2,143 | 2,350 | 3,285 | 2,160 |
| 3 | UV LMD | 1,530 | 18,996 | 1,473 | 8,308 | 8,006 | 15,491 | 3,334 | 2,450 | 10,625 | 2,673 |
| 3 | IR LCM | 1,742 | 12,173 | 999 | 7,201 | 6,021 | 24,101 | 2,687 | 1,874 | 8,342 | 2,167 |
| 4 | UV LMD | 546 | 11,048 | 2,535 | 54,176 | 5,193 | 2,890 | 2,497 | 3,558 | 6,130 | 3,194 |
| 4 | IR LCM | 457 | 7,817 | 1,466 | 21,786 | 3,889 | 3,093 | 1,438 | 1,343 | 2,497 | 1,990 |
Hunt et al., Table S1
